# Lessons from patients with hemoptysis attending a chest clinic in India

**DOI:** 10.4103/1817-1737.43062

**Published:** 2009

**Authors:** Rajendra Prasad, Rajiv Garg, Sanjay Singhal, Piyush Srivastava

**Affiliations:** *Department of Pulmonary Medicine, Chatrapati Sahuji Maharaj Medical University, Lucknow, India*

**Keywords:** Diagnosis, hemoptysis, mortality, treatment, tuberculosis

## Abstract

**OBJECTIVE:**

To evaluate the various etiologies of hemoptysis.

**MATERIALS AND METHODS:**

Four hundred and seventy-six consecutive patients of hemoptysis who were admitted to the Department of Pulmonary Medicine between January 1996 and December 2002 were included in this study. Hemoptysis was categorized as mild (< 100 ml/day), moderate (100–400 ml/day), and massive (>400 ml/day). We also categorized the patients according to the primary etiology of the hemoptysis.

**RESULTS:**

Of the 476 patients with hemoptysis included in this study, 352 were males and 124 were females. Pulmonary tuberculosis was the leading cause of hemoptysis. There were 377 (79.2%) patients in the pulmonary tuberculosis group, 25 (5.7%) in the neoplasm group, 19 (4.0%) in the chronic bronchitis group, 18 (3.8%) in the bronchiectasis group, and 35 (7.3%) patients with hemoptysis due to other causes. About one-third of the patients with hemoptysis had been misdiagnosed by the referring doctor as having active pulmonary tuberculosis.

**CONCLUSION:**

Although pulmonary tuberculosis is the most important cause of hemoptysis in India, it may also occur due to a variety of other causes. Awareness should be increased among general physicians about the various etiologies of hemoptysis in pulmonary tuberculosis patients.

Hemoptysis is defined as the expectoration of blood derived from the lungs or bronchial tree as a result of pulmonary or bronchial hemorrhage.[1] Hemoptysis is a frightening symptom for patients and often is a manifestation of a significant underlying disease such as bronchogenic carcinoma.[2] Expectoration of even a relatively small amount of blood is an alarming symptom, and massive hemoptysis can be a life-threatening event. Therefore, hemoptysis of any degree needs thorough evaluation.

Hemoptysis is a nonspecific symptom and can occur in about 100 different clinical conditions.[3] in India, hemoptysis is almost synonymous with pulmonary tuberculosis and patients presenting with this symptom are often prescribed antitubercular treatment without proper workup. The purpose of this study was to identify the various etiologies of hemoptysis, correct some common misconceptions regarding management of hemoptysis in tuberculosis, and to estimate the mortality due to hemoptysis.

## Materials and Methods

Four hundred and seventy-six consecutive patients of hemoptysis who were admitted to the Department of Pulmonary Medicine, Chatrapati Sahuji Maharaj Medical University (formerly King George's Medical College), Lucknow, India, between January 1996 and December 2002 were included in this prospective study. Approval from the institute's ethics committee and informed consent from all patients was obtained prior to commencement of the study. Demographic data, including sex and age, was collected. The amount of hemoptysis was also noted. Every patient was asked to collect the expectorated blood in a glass. The amount of hemoptysis was recorded and converted to a milliliter equivalent (i.e., one small glass = 100 ml). The episodes of hemoptysis were stratified into three groups according to the amount of blood expectorated, i.e., mild: < 100 ml/day; moderate: 100–400 ml/day; and massive: >400 ml/day.

Patients were also grouped into five broad categories on the basis of their primary diagnosis (i.e., pulmonary tuberculosis, neoplasm, chronic bronchitis, bronchiectasis, and ‘other’). Diagnosis was made after thorough clinical evaluation and appropriate investigations like chest radiography, computed tomography of thorax (CT), and bronchoscopy.

Diagnosis of pulmonary tuberculosis was based on chest radiography and sputum examination for acid fast bacilli. A chest radiograph demonstrating nodular, alveolar, or interstitial infiltrates predominantly affecting the upper zone of the lung(s) in symptomatic patients (i.e., those with cough, weight loss, and fever with night sweat) were considered suggestive of active pulmonary tuberculosis. When the chest radiograph showed inactive processes such as calcified granuloma, and there were no symptoms other than hemoptysis, the patient was diagnosed as having inactive pulmonary tuberculosis. For bronchogenic carcinoma, diagnosis was based on histopathology. Bronchitis was diagnosed when a patient had symptoms consistent with upper airway infection and a normal chest radiograph. Diagnosis of bronchiectasis was confirmed by high-resolution CT of thorax.

All patients were given conservative treatment for the control of hemoptysis, irrespective of the amount of blood expectorated, along with necessary measures for management of the primary disease. The conservative treatment comprised absolute bed rest, cough suppressant medications like codeine, mild sedation with alprazolam, antibiotics, and other supportive measures.

## Results

A total of 476 patients (352 males and 124 females) of hemoptysis were admitted in the hospital during the study period. The mean age of the patients was 35.5 ± 13.9 years. Based on the primary diagnoses, the patients were categorized into five main groups [Table 1]. Pulmonary tuberculosis was found to be the leading cause (79.2%) of hemoptysis in our patients. One hundred and forty-seven patients (30.9%) — 48 (10.1%) with inactive pulmonary tuberculosis and 99 (20.8%) with hemoptysis of non-tubercular etiology — had been misdiagnosed as active pulmonary tuberculosis and been prescribed antitubercular drugs before coming to our department.

**Table 1:** Etiology of hemoptysis

Primary diagnosis	Total number (%)
Pulmonary tuberculosis	377 (79.2)
Active	329 (87.3)
Inactive	48 (12.7)
Neoplasm	27 (5.7)
Primary (bronchogenic carcinoma)	24 (88.9)
Metastatic carcinoma	3 (11.1)
Chronic bronchitis	19 (4.0)
Bronchiectasis	18 (3.8)
Others	35 (7.3)
Idiopathic	13 (2.8)
Pneumonia	8 (1.7)
Cystic lung disease	4 (0.8)
Lung abscess	2 (0.4)
Larynx carcinoma	2 (0.4)
Cardiovascular	2 (0.4)
ABPA	1 (0.2)
SLE	1 (0.2)
TPE	1 (0.2)
Trauma	1 (0.2)

ABPA - Allergic bronchopulmonary aspergillosis, SLE - Systemic lupus erythematosus, TPE - Tropical pulmonary eosinophilia

Out of 329 patients of active tuberculosis, 52 patients (15.8%) had had an episode of hemoptysis during the course of their treatment and were referred to us as cases of multidrug resistant (MDR) tuberculosis. None of these 52 patients were found to have bacteriologically active tuberculosis.

Most of the patients in the study had moderate hemoptysis [Table 2]. Life-threatening hemoptysis was seen in only 5.3%. All the patients were treated conservatively with bed rest, cough suppressants, sedative and other supportive care. The bleeding stopped in 437(91.8%) of the 476 patients in 6.4 ± 6.0 days with conservative treatment alone. The remaining 39 patients died. Thus the overall mortality was 8.2%. Death was directly attributable to hemoptysis in only 25 (5.3%); these 25 patients were the ones who had had massive hemoptysis. In the rest (14 patients; 2.9%) death was due to the disease itself.

**Table 2:** Grading of hemoptysis in various disease

Grading	Tuberculosis	Lung cancer	Chronic bronchitis	Bronchiectasis	Others
Mild	19 (5.0)	24 (88.9)	11 (57.9)	13 (72.2)	26 (74.3)
Moderate	337 (89.4)	2 (7.4)	7 (36.8)	4 (22.2)	8 (22.9)
Massive	21 (5.6)	1 (3.7)	1 (5.3)	1 (5.6)	1 (3.8)
Total	377 (100)	27 (100)	19 (100)	18 (100)	35 (100)

Figures in parentheses are in percentages

## Discussion

Medical reviews from different part of the world revealed that the etiological pattern of hemoptysis has changed in the developed countries, with pulmonary tuberculosis is becoming less important as a cause of bleeding from the lungs.[4] In our country, however, the pattern has not changed. Pulmonary tuberculosis was the most common cause of hemoptysis four decades ago as shown by Rao in his study in 1960[5] and it is still the leading cause of it as is evident from the present study, in which tuberculosis was found in 79.2% of patients with hemoptysis. Various studies from other developing countries have also shown pulmonary tuberculosis to be the major cause of hemoptysis.[6–9] In our study, bronchogenic carcinoma was the second most common cause for the hemoptysis; some other Indian studies have not reported such a finding, probably because bronchogenic carcinoma could not be diagnosed due to poor diagnostic facilities and the cases were, therefore, included in the ‘undiagnosed’ category. Studies from developed countries have shown malignancy and non-tuberculous causes to be the leading reasons for hemoptysis.[10] In a retrospective study from United States, acute bronchitis was the most common cause of hemoptysis, followed by bronchogenic carcinoma.[11] The incidence of malignancy in various other studies from the developed world has ranged from 5–44%[12–18]; in comparison, in the present study it was 5.7%. The incidence of bronchiectasis seems to have declined when we compare the incidence found in the present study (3.8%) with the incidence reported by Rao[5] (13.6%) and Johnston,[12] (43%).

Occurrence of hemoptysis does not imply that active tuberculosis is present. Hemoptysis may occur as the initial manifestation of active tuberculosis, during the course of treatment, or even after the disease has been apparently cured. In patients with inactive pulmonary tuberculosis, only conservative (no anti-tuberculous drugs) management is necessary to control the bleeding. However, in our study, we found that antitubercular treatment had been prescribed by general physicians to all the patients of inactive pulmonary tuberculosis. Conservative management plus continuation of antitubercular treatment can control hemoptysis in patients having an episode during their course of treatment. Hemoptysis occurring in a patient during the course of treatment (of tuberculosis) does not mean that the patient is not responding or that the infection is drug resistant. In our study, out of 329 patients with active tuberculosis, 52 patients (15.8%) had episodes of hemoptysis during the course of their treatment and were referred to us as cases of MDR tuberculosis. This may be due to lack of awareness among general physicians about the various causes of hemoptysis in pulmonary tuberculosis patients.

The overall mortality rate of 8.2% in this study is somewhat lower than that reported by Knott-Craig *et al.*[19] in a study of 120 adult patients (overall mortality rate: 10%) and Corey *et al.*[20] in a report of 59 adult patients (mortality rate: 9%). This difference may be due to differences in the study groups. In our study group, most of the patients had moderate hemoptysis, whereas in the other studies most patients had massive hemoptysis. Several studies in adults have shown that the mortality increases with the amount of hemoptysis.[21–23]

Based on the above study, we came to the following conclusions:

1. Pulmonary tuberculosis is still the major cause of hemoptysis in developing countries like India.
2. Hemoptysis does not always reflect underlying pulmonary tuberculosis. Hence antitubercular treatment should not be started without proper diagnostic workup.
3. Hemoptysis can occur during the course of antitubercular treatment; hemoptysis in a case of pulmonary tuberculosis, which otherwise shows clinical, radiological, and bacteriological improvement, does not always indicate drug-resistant tuberculosis.
4. Hemoptysis does not warrant the initiation of antitubercular treatment in a successfully cured patient who does not show any clinico-radio-bacteriological evidence of active pulmonary tuberculosis.

One limitation of our study was that due to nonavailability of advanced techniques in our hospital, we could not provide optimum care for patients with massive hemoptysis.
